# A new biocompatible ternary Layered Double Hydroxide Adsorbent for ultrafast removal of anionic organic dyes

**DOI:** 10.1038/s41598-019-52849-4

**Published:** 2019-11-07

**Authors:** Garima Rathee, Amardeep Awasthi, Damini Sood, Ravi Tomar, Vartika Tomar, Ramesh Chandra

**Affiliations:** 1 Drug Discovery & Development Laboratory, Department of Chemistry, University of Delhi, Delhi, 110007 India; 2 Dr. B. R. Ambedkar Centre for Biomedical Research University of Delhi, Delhi, 110007 India

**Keywords:** Pollution remediation, Materials chemistry

## Abstract

It would be of great significance to introduce a new biocompatible Layered Double Hydroxide (LDH) for the efficient remediation of wastewater. Herein, we designed a facile, biocompatible and environmental friendly layered double hydroxide (LDH) of NiFeTi for the very first time by the hydrothermal route. The materialization of NiFeTi LDH was confirmed by FTIR, XRD and Raman studies. BET results revealed the high surface area (106 m^2^/g) and the morphological studies (FESEM and TEM) portrayed the sheets-like structure of NiFeTi nanoparticles. The material so obtained was employed as an efficient adsorbent for the removal of organic dyes from synthetic waste water. The dye removal study showed >96% efficiency for the removal of methyl orange, congo red, methyl blue and orange G, which revealed the superiority of material for decontamination of waste water. The maximum removal (90%) of dyes was attained within 2 min of initiation of the adsorption process which supported the ultrafast removal efficiency. This ultrafast removal efficiency was attributed to high surface area and large concentration of -OH and CO_3_^2−^ groups present in NiFeTi LDH. In addition, the reusability was also performed up to three cycles with 96, 90 and 88% efficiency for methyl orange. Furthermore, the biocompatibility test on MHS cell lines were also carried which revealed the non-toxic nature of NiFeTi LDH at lower concentration (100% cell viability at 15.6 μg/ml). Overall, we offer a facile surfactant free method for the synthesis of NiFeTi LDH which is efficient for decontamination of anionic dyes from water and also non-toxic.

## Introduction

The organic dye contaminants released from various sources are one of the major global environmental issues^1^. The organic water-soluble dyes can be classified as anionic, cationic and non-ionic dyes^2^. Most of these dyes have been reported to be mutagenic, toxic and cancer causing in nature^3^. Unfortunately, 7 × 10^5^ tons of dye contaminants are released into water every year which has posed a threat to aquatic life by reducing the penetration of light and visibility through the water surface. This has a direct consequence on the photosynthetic cycle^4^.

To study the effect of dyes on the ecosystem a number of studies are performed for example the effects of five azo food dyes (tartrazine, carmoisine, sunset yellow, ponceau 4R and allura red) on the *in vitro* synthesis of leukotriene B_4_ and F_2_-isoprostanes and suggested their high potency to promote pro-inflammatory responses^5^. Oliveria *et al*. used ecotoxicity testing battery to study the genotoxicity and acute toxicity of various dyes on different tropic levels. They landed up with the conclusion that Direct Black 38 and Reactive Blue 15 dyes causes acute toxicity and genotoxicity among aquatic organisms. However, Direct Black 38 was considered to be most toxic dye because of its lethal toxicity and tendency for DNA damage^6^. Further, molecular docking and multispectroscopy studies were also carried out by Naveenraj *et al*. to study the interaction of Acid Orange 10 with Bovine Serum Albumin (BSA) and Human Serum Albumin (HSA) and therefore, revealed the toxic effects of Acid Orange 10 during blood transportation process^7^. As a consequence, the need of the hour is to develop an effective technique for the removal of dye contaminants from wastewater.

Various techniques like biological treatment^8^, coagulation^9^, ozonation^10^, membrane filtration^11^, chemical oxidation^12^, ion exchange methods^13^, photocatalysis^14,15^ and adsorption^16^ have been widely used for dye removal but they suffer various limitations. Among all the techniques, the adsorption process is the most widely adopted and reliable technique because of its high productivity, assessibility, economic feasibility, recyclability and reuse of adsorbents and the vast choice of adsorbents^9,17^. A large number of adsorbents have been advocated for the wastewater remediation. One of the adsorbents is activated carbon, but it is not economically desirable^18,19^. Other alternative candidates are fly ash^17^, polymers^19^, zeolites^20^ and clays^21^. Among all the known adsorbents, layered double hydroxides (LDHs) have been receiving consistent recognition because they offer diverse advantages such as great adsorption capacity, economically feasible and high anion-exchange properties^22^.

LDHs, also known as anionic clays, are extensively used in the field of catalysts and anion exchanges^23,24^, adsorbents^25^, anticorrosion agents^26^, drug and catalyst carriers^27^ and electrode materials^28,29^. The general formula of LDH, an important inorganic layered material is [M_(1-x)_^II^M_x_^III^(OH)_2_]^x+^(A^n−^)_x/n_.mH_2_O, where M^II^ and M^III^ are divalent (Cu^2+^, Ca^2+^, Mg^2+^, Zn^2+^, Ni^2+^, Co^2+^, etc.) and trivalent metal ions (Al^3+^, Fe^3+^, Ga^3+^, Cr^3+^, etc.), A^n-^(Cl^−^, Br^−^, NO_3_^−^, I^−^, OH^−^, SO_4_^2−^, etc.) is the interlayer anion with charge n and x is the molar ratio between divalent and trivalent cations M^3+^/(M^2+^ + M^3+^)^29–33^. LDH materials having a wide range of physicochemical properties can be designed by various methods such as co-precipitation^34^, ion-exchange methods^35^, hydrothermal methods^36^, urea hydrolysis^37^, ultrasound irradiation^38^ and rehydration/reconstruction^39^. The presence of replaceable interlayer anions makes LDHs good anion-exchangers.

To enhance the adsorbent properties of LDHs, scads of variations have been introduced by the researchers across the globe, such as MgAl-LDH supported Cu-(BDC) MOF^40^, Ni_4_Fe_1_-Cl-LDH^41^, ZnAl-LDH/Al(OH)_3_ ^42^, etc (Table 1) but unfortunately they cannot be applied under high acidic or high basic conditions, exhibits toxic nature and display slow adsorption rate, thus, making them inapplicable for wastewater treatment. So, it would be of great significance to introduce a new biocompatible, environment friendly and ultrafast adsorbent for wastewater management.

**Table 1:** Reported adsorbents for dye removal and their parameters.

Adsorbent	Dye implemented	% Removal (approx.)	Contact time (approx.)	pH	Reference
MgAl-LDH supported Cu-(BDC) MOF	Methyl orange	99	20 min	6	^40^
Ni4Fe1- CO_3_-LDH	Methyl orange	20	120 min	5–6	^41^
ZnAl-LDH/Al (OH)_3_	Methyl Orange	98	50 min	4	^42^
ZnAl-LDH/Al (OH)_3_	Congo Red	99	200 min	6	^42^
S/NiFe-LDH (1:1),	Methyl Orange	82.6	30 min	3	^43^
S/NiFe-LDH (2:1)	Methyl Orange	63.2	60 min	3	^43^
Mg−Al−CO_3_ LDH− carbon dot composites	Methyl blue	96	20 min	9.45	^44^
C/MnCuAl LDOs	Congo Red	Attained equilibrium (58%)	180 min	4.5	^45^
Ca-Al-LDHs	Congo Red	Attained equilibrium	80 min	7	^46^
Ni/Fe/Ti LDH Ni/Fe/Ti LDH	Methyl Orange	>95%	4 min	7–8	In this study
	Congo Red	>95%	6 min	5–12	In this study
Ni/Fe/Ti LDH	Methyl blue	>95%	1 min	5–12	In this study
Ni/Fe/Ti LDH	Orange G	>95%	4 min	7–8	In this study

Our study demonstrates the highly effective ultrafast adsorption of different organic dye contaminants over ternary Ni/Fe/Ti LDH in comparison to the previous studies^40–46^. Here, we have reported the synthesis of a series of ternary Ni/Fe/Ti LDHs with varying Ni/Fe/Ti ratio via the hydrothermal route. The synthesized material was implemented for the dye adsorption from synthetic wastewater. The biocompatible study of synthesized Ni/Fe/Ti LDH was also carried out using MHS cell lines with varying concentrations of Ni/Fe/Ti LDH which confirmed the non-toxic behavior of synthesized rapid adsorbent and open all the channels for implementing it in water remediation.

## Experimental Section

### Materials

All chemicals were commercially available and were used without further purification. Fe(NO_3_)_3_.9H_2_O and TiCl_4_ were purchased from LOBA CHEMIA (Mumbai, India). Ni(NO_3_)_2_.6H_2_O was purchased from THOMAS BAKER (Mumbai, India). Methyl orange, congo red, methyl blue, orange G, methylene blue and rhodamine B were purchased from SIGMA-ALDRICH (USA). HCl was purchased from FISCHER SCIENTIFIC (Mumbai, India). NaOH was purchased from spectrochem. For biocompatibility, RPMI-1640 media, trypsin, Phosphate Buffer Saline (PBS), Fetal Bovine Serum (FBS), Dimethyl Sulfoxide (DMSO),3-(4,5-Dimethylthiazol-2-Yl)-2,5-Diphenyltetrazolium Bromide (MTT) dye were procured from himedia. MHS (Mouse macrophagic) cell line was procured from National Centre For Cell Science, Pune.

### Synthesis of LDHs

Ni/Fe/Ti LDHs (NiFeTi1, NiFeTi2, NiFeTi3, NiFeTi4 and NiFeTi5) were synthesized by the hydrothermal route with varied concentrations. The synthesis was carried out by adding Ni(NO_3_)_2_.6H_2_O, Fe(NO_3_)_3_.9H_2_O, TiCl_4_ and 1.5 g urea in 100 ml decarbonated water. The mixture was vigorously stirred and hydrothermally aged in an autoclave at 160 °C for 2 days. The product so formed was washed with distilled water and dried in an oven at 80 °C. (**S2** – Electronic supplementary information)

### Biocompatibility studies

MHS cell lines were grown in RPMI-1640 media in 5% CO_2_ incubator at 37 °C. When the cells reached 70% confluency, they were trypsinized and harvested. Cells were counted on a hemocytometer. 10,000 cells were seeded per well on a 96 well plate. Cells were left to attach for another 24 h. Different concentration of NiFeTi2-LDH (15 μg/ml, 31.5 μg/ml, 62.5 μg/ml and 125 μg/ml) were added to it and left for 24 h. Finally, media was pipetted out gently and 0.5 mg/ml MTT dye was added per well. After 4 h of incubation DMSO was added and left for 10 min to solubilize the formazan crystal. Absorbance was taken at 570 nm with 630 nm reference.

### Characterization methods

X-ray diffractometer (Model No. D8 DISCOVER) was used to record X-ray diffraction patterns. Morphology of the synthesized material was studied using TECNAI 200 kV Transmission Electron Microscopy (TEM) (Fei, Electron Optics) and Field Emission Scanning Electron Microscope (FESEM) Model No.- ZEISS Gemini SEM-500. The Raman spectra were obtained from RenishawinVia^TM^ Reflex Micro-Raman spectrometer using 514 nm wavelength Ar^+^ laser for sample excitation. TGA spectra were recorded on LINSEIS L40/2052. FT-IR spectra were recorded on Thermo Scientific Nicolet iS 50 FTIR. UV-vis spectra were measured on Thermo Scientific Evolution 300. BET was recorded on Quantacrome Instruments Autosorb-1.

### Adsorption experiments

All the adsorption experiments were performed in batches by dispersing adsorbent (20 mg) in10 mL dye solution (20 mg/L) for 10 min (pH = actual, temperature = 25 °C). The adsorbent was further separated by centrifugation and the solution was used to evaluate the residual dye concentration by using UV-vis spectrometer. The effect of contact time, pH, amount of the adsorbent and the initial concentrations of the dye on the adsorption process were also evaluated. The adsorption kinetics was studied at time intervals of 1, 2, 4, 6, 8 and 10 min. The adsorption isotherm was studied by varing the initial dye concentrations (ranging from 10–70 mg/L).

The dye removal efficiency (DRE) was calculated by the formula:

1 $${\rm{Dye}}\,{\rm{removal}}\,{\rm{efficiency}}\,( \% )=[({{\rm{C}}}_{0}-{{\rm{C}}}_{{\rm{e}}})/{{\rm{C}}}_{0})]\times 100 \% $$

The adsorption capacity at equilibrium, q_e_ (mg/g) was evaluated by the equation:

2 $${\rm{Adsorption}}\,{\rm{capacity}}\,({{\rm{q}}}_{{\rm{e}}})=({{\rm{C}}}_{0}-{{\rm{C}}}_{{\rm{e}}})\times {\rm{V}}/{\rm{W}}$$

The adsorption capacity at time t, q_t_ (mg/g) was calculated by:

3 $${\rm{Adsorption}}\,{\rm{capacity}}\,({{\rm{q}}}_{{\rm{t}}})=({{\rm{C}}}_{0}-{{\rm{C}}}_{{\rm{t}}})\times {\rm{V}}/{{\rm{W}}}^{3}$$

where, C_0_ is the initial concentration of dyes, C_e_ is the equilibrium concentration of dyes and C_t_ is the concentration of dyes at respective time interval. Reaction mixture volume is denoted by V (L) and adsorbent amount used is represented by W (g).

## Result and Discussion

### Characterization

**Figure 1:**
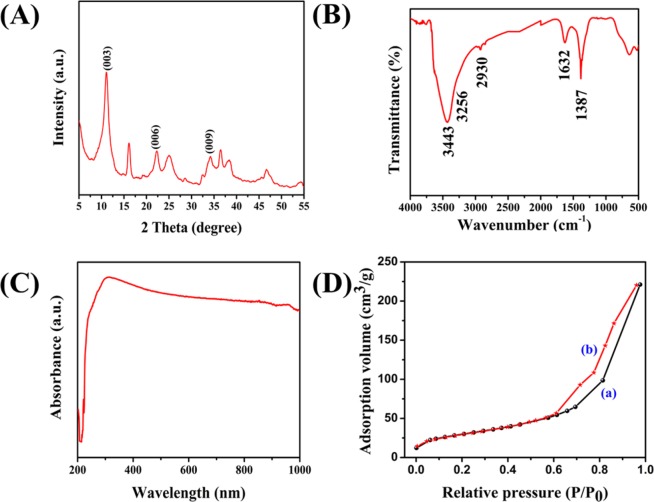
(**A)** XRD Pattern of NiFeTi2 LDH, (**B)** FTIR spectra of NiFeTi2 LDH, (**C)** UV-vis spectroscopy of NiFeTi2 LDH, (**D)** (a) N_2_-adsorption and (b) N_2_-desorption of NiFeTi2 LDH.

The XRD pattern of NiFeTi2 LDH can be easily correlated with previously reported data^47^. The incorporation of CO_3_^2−^ ions and H_2_O molecules in the lattice of synthesized LDH can be confirmed by the 2θ values of 11.12, 22.32 and 34.31° representing (003), (006) and (009) reflections, respectively. The d-spacing of the planes (003) and (110) were found to be 0.796 nm and 0.354 nm, respectively. Due to the similarity in the basal spacing of the synthesized LDH and Ti incorporated LDHs, it could be easily stated that the interlayer CO_3_^2−^ ions and H_2_O molecules have retained a pattern that is similar to the previously reported XRD patterns. Moreover, the presence of (110) and (101) reflections at 25.1° and 36.5° indicates that TiO_2_ exists in anatase phase in the synthesized LDH. The XRD pattern of NiFeTi2 LDH is illustrated in Fig. 1(A), XRD patterns of all the synthesized materials is depicted in Fig. S3a(A) and X-ray diffraction parameters are illustrated in Table S1.

During the variation in the prescribed synthesis of LDH, we have performed the PXRD experiment by which we came to observed that the composition of LDH is in its purest form. Simultaneously, we have observed that other material excluding NiFeTi2 LDH contains different oxides other than LDH. Therefore, we have analysed the ICP results of different as-synthesized LDH materials. The pure LDH synthesised as NiFeTi2 LDH (Ni_2.49_Fe_0.2_Ti_1.0_) and other LDH chemical compositions, with intercorporated CO_3_^2−^ ions and H_2_O molecules, has been provided in Table 2.

**Table 2:** Kinetic parameters obtained from the kinetic study.

Dye	Pseudo-first-order			Pseudo-second-order		
	q_e_ (mg/g)	k_1_ (min^−1^)	R^2^	q_e_ (mg/g)	k_2_ (g/mg min)	R^2^
MO	8.800	0.584	0.994	10.108	0.220	0.993
CR	4.664	0.243	0.646	10.121	0.268	0.994
MB	1.359	0.432	0.452	9.798	2.934	0.999
OG	3.886	0.616	0.777	10.08	0.943	0.999

FTIR spectra of all the synthesized materials are depicted in Fig. S3a(B) and spectrum of NiFeTi2 LDH is depicted in Fig. 1(B). The broad absorption band located around 3443 cm^−1^ might be due the stretching vibration occurred because of the presence of layer’s hydroxyl group and intercalated water molecules. The shoulders around 3256 cm^−1^ and 2930 cm^−1^ could be due to the H-bonding arising between the interlayer H_2_O and CO_3_^2−^ anions. A weaker band at 1632 cm^−1^ could be assigned to the bending vibrations of hydroxyl group. The observation band at 1387 cm^−1^ could be asymmetric stretching occurred due to the existence of carbonate ions^48,49^.

The UV-visible spectra of all the synthesized materials are illustrated in Fig. S3a(C). The coordination state and the nature of Ni, Fe and Ti within the layered framework is introduced by the UV-vis of the various LDHs. The hump at ≈230 nm, observed for all the LDHs is attributed to the d-d transitions. The strong adsorption band observed in the range of 250–460 nm can be assigned to typical metal coordinated to the CO_3_^2−^ anion present in the interlayer galleries^47,48,50–55^. A broad shoulder between 385 to 700 nm may be attributed to the existence of Ti^4+^ in the brucite-like sheets (Fig. 1(C))^47^. Broad valley also signifies supramolecular guest-host interactions (hydrogen bonding, electrostatic attraction and van der Waals forces) or guest-guest interactions (van der Waals forces and hydrogen bonding).

The N_2_ adsorption- desorption isotherm of NiFeTi2 is depicted in Fig. 1(D). The isotherm of NiFeTi2 LDH could be easily correlated with H-3 Type hysteresis loop. The hysteresis loop appearing at 0.6–1, suggests the presence of mesoporous material. The calculated BET surface area and Langmuir surface area were 106 m^2^/g and 1702 m^2^/g for NiFeTi2 LDH, respectively. The average pore diameter was also determined by using Barrett-Joyner-Halenda (BJH) method and is obtained as 10.6 nm. The BJH adsorption and desorption pore size distribution volumes were reported as 0.33 and 0.34 cm^3^/g, respectively^49,56^.

**Figure 2:**
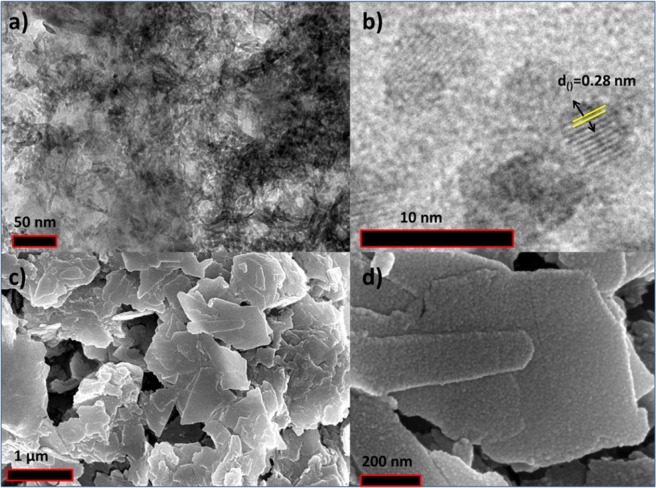
(**a**,**b**) HRTEM images of NiFeTi2 LDH; (**c**,**d**) FE-SEM images of NiFeTi2 LDH.

In addition, SEM images (Fig. 2(c,d)) of CO_3_-LDH show platelet structure. The HRTEM images (Fig. 2(a,b)) clearly indicates the sheets-like morphology of CO_3_-LDH with d-spacing of 0.27 nm.

**Figure 3:**
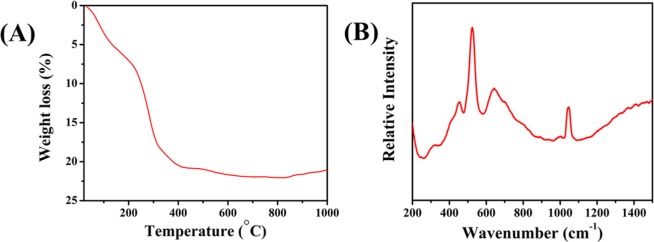
(**A)** TGA spectra of NiFeTi2 LDH, (**B)** Raman spectra of NiFeTi2 LDH.

The thermogram (Fig. S3b(A)) of the synthesized materials exhibit two degradation steps. The first degradation occurs from 50–200 °C due to the elimination of surface and interlayer water, followed by second degradation (250–400 °C) due to the decarbonation and dehydroxylation (Fig. 3(A))^48^.

Figures 3(B) and S3b(B) shows the Raman studies of carbonate anions in NiFeTi2 and all LDHs, respectively. In all the LDHs, ν_1_ band is observed around 1038 cm^−1^. In NiFeTi1, ν_1_ is a weak band which becomes stronger in the case of NiFeTi2, which further becomes weak and broad in the case of NiFeTi3 and NiFeTi4 and finally disappears in the case of NiFeTi5. It is also observed that ν_4_ band is weakest in NiFeTi1 at 628.28 cm^−1^, stronger in NiFeTi2 at 639.1 cm^−1^, more stronger for NiFeTi3 (639.14 cm^−1^), broader and stronger in NiFeTi4 at 655.42 cm^−1^ and broadest and strongest for NiFeTi5. These observed trends in ν_4_ bands is due to the overlap of ν_4_ band of CO_3_^2−^ and M-OH of hydroxide layer as interlayer anions and this vibration is assigned to the Eg ($\tau$) mode. These two bands overlap result in broadening of ν_4_ band.

### Adsorbent variation for adsorption study

**Figure 4:**
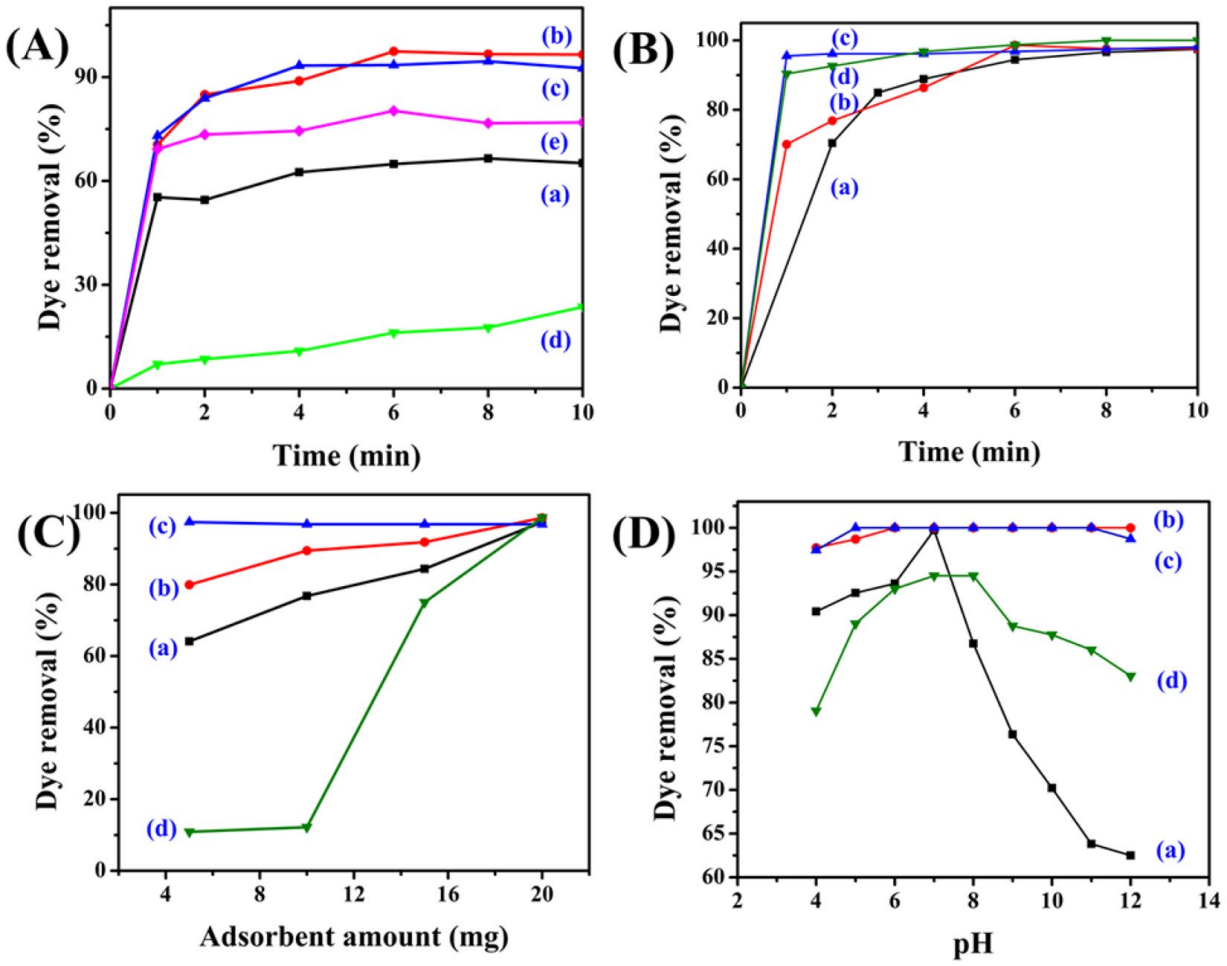
(**A**) Dye removal (%) of MO over various LDHs (Reaction conditions: adsorbent amount = 20 mg, V_solution_ = 10 mL, conc of MO = 20 mg/L, actual pH, temperature = 25 °C) [(a) NiFeTi1, (b) NiFeTi2, (c) NiFeTi3, (d) NiFeTi4, (e) NiFeTi5], (**B)** Influence of contact time over *NiFeTi2 LDH* (Reaction conditions: adsorbent amount = 20 mg, dye solution = 10 mL, conc of dye solution = 20 mg/L, pH = 7.35, temperature = 25 °C) [(a) MO, (b) CR, (c) MB, (d) OG], (**C)** Influence of adsorbent amount over *NiFeTi2 LDH* (Reaction conditions: V_solution_ = 10 mL, conc of dye solution = 20 mg/L, time = 10 min, pH = 7.35, temperature = 25 °C) [(a) MO, (b) CR, (c) MB, (d) OG], (**D)** Influence of pH on dye removal (%) of various anionic dyes over *NiFeTi2 LDH* (Reaction conditions: adsorbent amount = 20 mg, V_solution_ = 10 mL, conc of dye solution = 20 mg/L, time = 10 min, temperature = 25 °C) [(a) MO, (b) CR, (c) MB, (d) OG].

Initially, MO was used to carry out the adsorption experiment aiming to select the optimum adsorbent from the series, NiFeTi1-NiFeTi5. The dye removal efficiency of each adsorbent was calculated, shown in (Fig. 4(A)). Among all adsorbents (NiFeTi1-NiFeTi5) implemented for adsorption study, the ternary NiFeTi2 which is purely LDH as compared to all other materials showed the highest adsorption capacity with 97.44% removal of anionic dye from aqueous solution and attained equilibrium between 4 to 6 min. This might be due to the inherit properties associated with pure LDH, mainly which are layered structure, large surface area and interlayer ion exchange^43^. Along with this, many factors counting electronegativity, electrostatic attractions and hydrogen bonding might be responsible behind such efficient adsorption. A promising mechanism behind such adsorption could be the formation of H-bonds among the dye molecules and –OH groups of NiFeTi2 LDH. Also, the formation of electrostatic attraction between negative and positive charges of dye molecules and NiFeTi2 LDH surface, respectively, results in excellent adsorption of anionic dyes^48^.

### Dye variation for adsorption study

**Figure 5:**
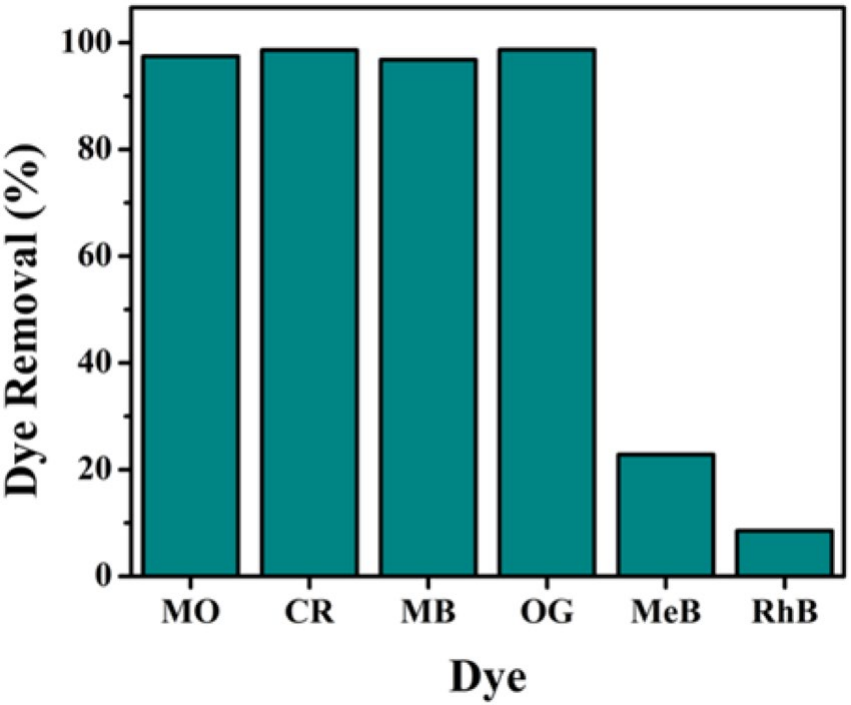
Removal efficiency of *NiFeTi2 LDH* for various organic dyes (Reaction conditions: adsorbent amount = 20 mg, V_solution_ = 10 mL, conc of dye solution = 20 mg/L, time = 10 min, temperature = 25 °C, pH = 7.35).

The adsorption studies for various organic dyes over NiFeTi2 LDH were performed. It was observed from Fig. 5 that the anionic dyes (MO (97.44%), CR (98.63%), MB (96.81%) and OG (98.71%)) are adsorbed more preferentially over the LDH surface than cationic dyes (MeB (22.81%) and RhB (8.5%)). Moreover, the low adsorption value for cationic dyes might be because of the unfavourable resistance of positive charge present in cationic dyes from the positively charged surface of the LDH on contrary to anionic dyes. Therefore, due to the presence of opposite charge, attraction electrostatic forces took place for the adsorption of the anionic dyes and enhanced their adsorption drastically on the LDH surface. Further studies were conducted using anionic dyes (MO, CR, MB and OG). The UV-visible spectra of time dependent adsorption of various dyes over NiFeTi2 LDH are depicted in Fig. S4.

### Effects of various parameters (contact time, adsorbent dosage, pH of the solution and initial dye concentration)

The influence of contact time on dye removal (%) of MO, CR, MB and OG over NiFeTi2 LDH is shown in Fig. 4(B). For all dyes, adsorption was achieved within 10 min from the initial start of the process which might be due to the existence of easily available active sites on the exterior surface of NiFeTi2 LDH^40–46^. Further, the effect of alteration in the adsorbent amount (5 to 20 mg) on the dye removal efficiency was evaluated (Fig. 4(C)). It was observed that when the amount of the adsorbent was increased the dye removal (%) also increases. Hence, from the experimental data 20 mg adsorbent amount was chosen for further evalution.

Figure 4(D) demonstrates the cleaning efficiency of NiFeTi2 LDH over a wide range of pH = 4 to 12 (Solutions used for adjusting pH = 0.1 M HCl, 0.1 M NaOH). The results showed that the dye removal efficiency of NiFeTi2 LDH was highest at pH 7 (99.73% (MO), 100% (CR), 100% (MB) and 94.51% (OG)). 100% dye removal efficiency is shown by adsorbent for CR and MB at each and every pH. As per the results suggested, the effect of pH change is noteworthy and had given very indicative information for the dye and adsorbent interactions. For lower pH (pH < 7), the adsorbent is prone to the dissolution which results in adsorption and less availability of active adsorbent sites. Henceforth, dissolution of LDH at lower pH has decreased removal efficiency of dyes. According to study by Ai *et al*., 2011, the negative charge on the adsorbent surface keeps on increasing with increase in value of pH and results in electrostatic repulsion between the anionic organic dye molecules and adsorbent surface. Therefore, in contrast to lower pH, the lower dye removal percentage at higher pH can be attributed the purely electrostatic force of repulsion^1^.

**Figure 6:**
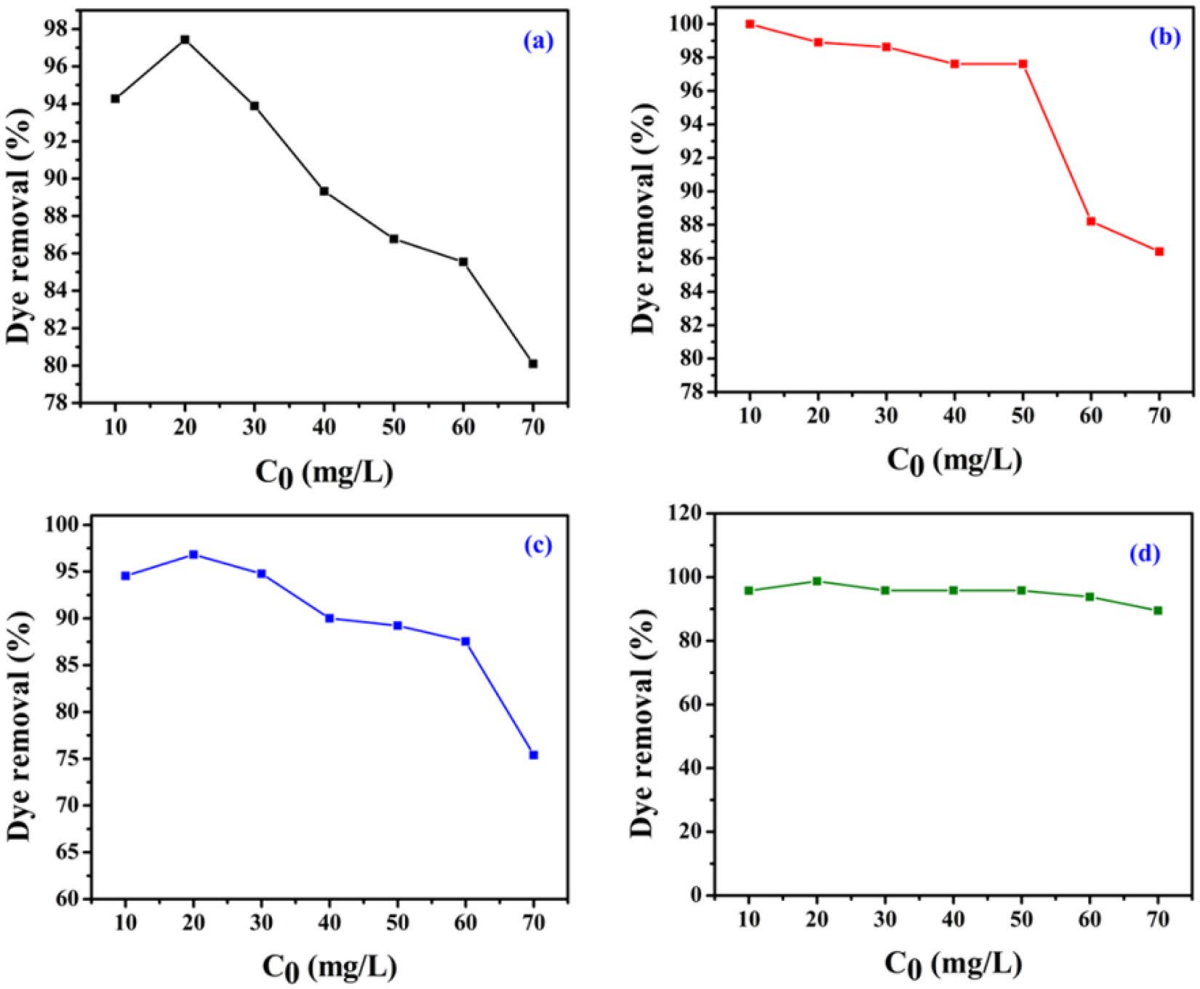
Dye removal (%) efficiency of *NiFeTi2 LDH* for various initial dye concentrations dyes [(**a**) MO, (**b**) CR, (**c**) MB, (**d**) OG] (V_solution_ = 10 mL, adsorbent amount = 20 mg, T = 25 °C, Time = 10 min, pH = 7.35).

Figure 6 depicts the variation in the removal efficiency of NiFeTi2 LDH observed when the initial dye concentration was varied from 10–70 ppm. The results clearly demonstrated a gradual decrease in removal efficiency when dye concentration was increased. The dye removal efficiency decreases from 97.44 to 80.1%, 100 to 86.39%, 96.81 to 75.4% and 98.71 to 89.46%, for MO, CR, MB and OG, respectively. This change could be attributed to the change in the ratio of the active adsorbent sites to the number of dye molecules with increase of dye concnetration^49^.

### Kinetics study

**Figure 7:**
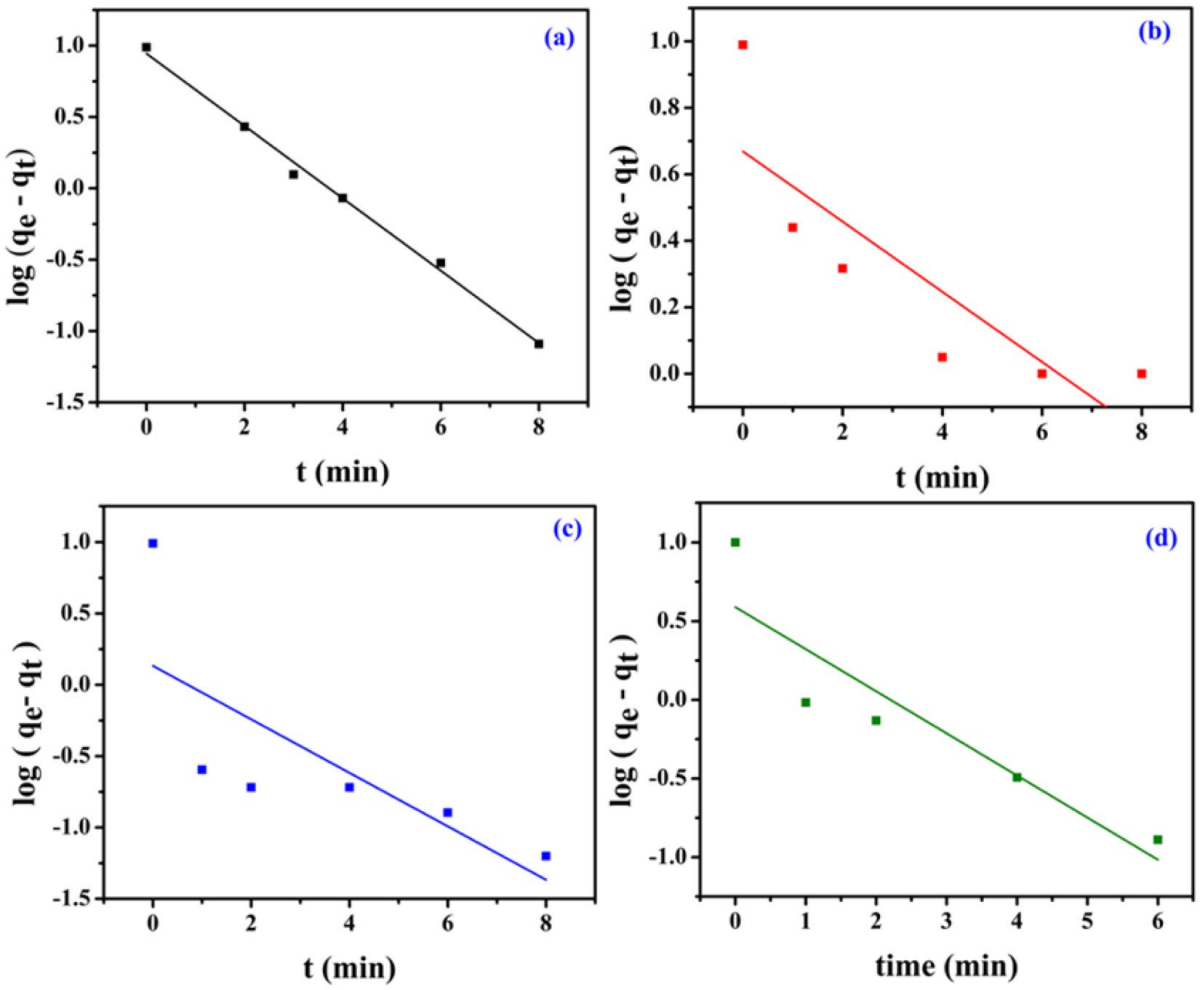
Kinetic plots (Pseudo-first order) for (**a**) MO, (**b**) CR, (**c**) MB and (**d**) OG adsorption (V_solution_ = 10 mL, C_0_ = 20 ppm, adsorbent amount = 20 mg, T = 25 °C, pH = 7.35).

**Figure 8:**
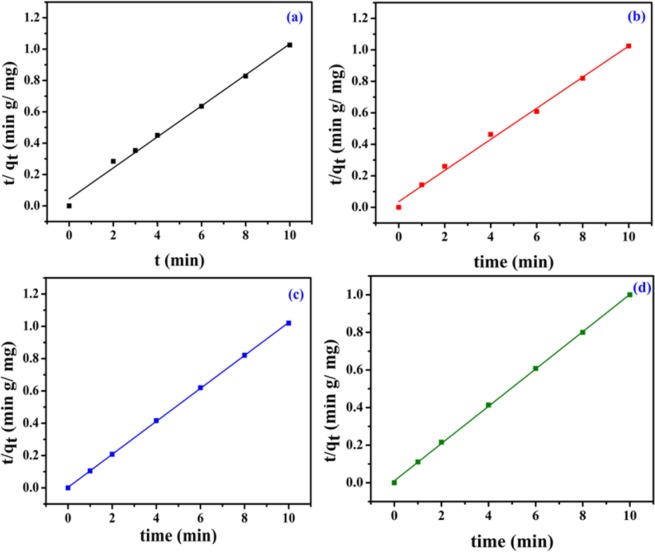
Kinetic plots (Pseudo-second order) for (**a**) MO, (**b**) CR, (**c**) MB and (**d**) OG adsorption (V_solution_ = 10 mL, C_0_ = 20 ppm, adsorbent amount = 20 mg, T = 25 °C, pH = 7.35).

Pseudo-first and second-order kinetic models are used for kinetic studies of the removal of various dyes by NiFeTi2 LDH. The models are represented as

4 $$\log ({{\rm{q}}}_{{\rm{e}}}\mbox{--}{{\rm{q}}}_{{\rm{t}}})=\,\log \,{{\rm{q}}}_{{\rm{e}}}\mbox{--}\frac{{{\rm{k}}}_{1}}{2.303}{\rm{t}}$$

5 $$\frac{{\rm{t}}}{{{\rm{q}}}_{{\rm{t}}}}=\frac{1}{{{\rm{k}}}_{2}{{\rm{q}}}_{{\rm{e}}}^{2}}+\frac{1}{{{\rm{q}}}_{{\rm{e}}}}{\rm{t}}$$

6 $${{\rm{q}}}_{{\rm{t}}}={{\rm{k}}}_{{\rm{i}}}{{\rm{t}}}^{0.5}+{\rm{C}}$$

where, q_e_ (mg/g) and q_t_ (mg/g) are the amount of dye adsorbed at equilibrium and at time (t), respectively; k_1_ (min^−1^), k_2_ (g/mg min) and k_i_ (g/mg min^0.5^) are the pseudo-first-order, pseudo-second-order and intraparticle diffusion rate constants, respectively and C is the intercept. The pseudo-first-order and the pseudo-second-order kinetic model fittings are illustrated in (Figs 7 and 8), and their parameters q_e_, k_1_ and correlation coefficient (R^2^) are provided in Table 2.

From the linear form of pseudo-first order kinetic model (Eq. 4), the values of k_1_ and q_e_ were estimated from the slope and intercept of linear plots [log(q_e_ – q_t_) versus t] (Fig. 7). Similarly, from the linear form of pseudo-second order kinetic model (Eq. 5), the values of k_2_ and q_e_ were evaluated (Fig. 8). All the kinetics parameters are summarized in Table 2. In all the cases, it was observed that the adsorption process followed pseudo-second order kinetic model with higher correlation coefficient (R^2^) value as compared to first order kinetic model.

**Figure 9:**
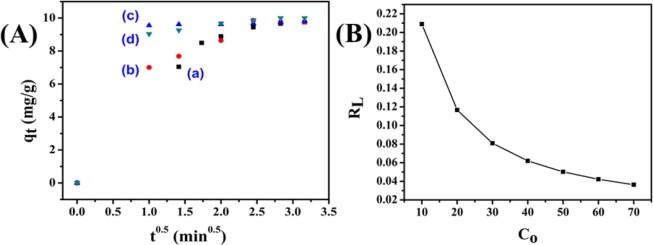
(**A)** Intraparticle diffusion kinetic plot for adsorption of (a) MO, (b) CR, (c) MB and (d) OG over NiFeTi2 LDH (V_solution_ = 10 mL, C_0_ = 20 ppm, adsorbent amount = 20 mg, T = 25 °C, pH = 7.35), (**B)** R_L_ v/s C_0_ plot for adsorption of MO over NiFeTi2 LDH.

Using the intraparticle diffusion kinetic model (Eq. 6), the diffusion mechanism of the adsorption process was investigated. The value of k_i_ has been calculated by using the slope and C is the intercept of the plots of q_t_ versus t^0.5^ (Fig. 9(A)). Here, the intercept C refers to the boundary layer thickness. For all the cases, it is observed that the adsorption process has been influenced by multiple processes. From the plots, it could be inferred that along with intraparticle diffusion mechanism another mechanism could also be responsible for the process^1^. The initial portion of the plot signifies the adsorption on the outer surface of NiFeTi LDH, the second portion of the graph indicates the pores adsorption (intraparticle diffusion) and the unavailability of the free adsorptive sites after reaching the equilibrium stage is indicated by the third portion of the plot^1,49^.

### Adsorption isotherm

**Figure 10:**
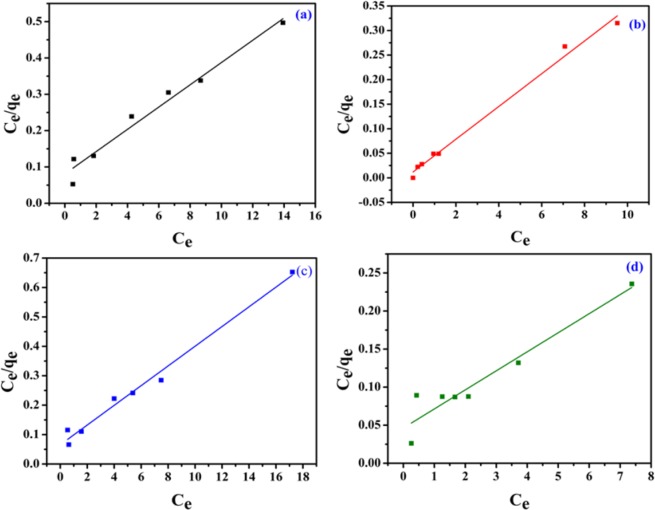
Isotherm plots (Langmuir model) for (a) MO, (b) CR, (c) MB and (d) OG adsorption (V_solution_ = 10 mL, adsorbent amount = 20 mg, T = 25 °C, pH = 7.35).

Adsorption isotherms are the route to get qualitative information of the adsorption capacity of adsorbents and the distribution of the adsorbates between liquid and solid phases after reaching equilibrium. Langmuir and Freundlich isotherm models are used to study the adsorption capacity of the adsorbent at different equilibrium concentrations. The linear representation of the Langmuir and Freundlich isotherms are

7 $$\frac{{{\rm{C}}}_{{\rm{e}}}}{{{\rm{q}}}_{{\rm{e}}}}=\frac{1}{{{\rm{q}}}_{{\rm{m}}}{\rm{b}}}+\frac{{{\rm{C}}}_{{\rm{e}}}}{{{\rm{q}}}_{{\rm{m}}}}$$

where, C_e_ (mg/L) is the equilibrium concentration of the adsorbate, q_e_ (mg/g) is the equilibrium adsorption capacity of the adsorbent, q_m_ (mg/g) is the maximum adsorption capacity of the adsorbent and b (L/mg) is the Langmuir constant. The linear plot of C_e_/q_e_ versus C_e_ (Eq. 7) gives the values of q_m_ and b depicted in (Fig. 10) and results are summarized in Table 3. The adsorption results illustrated higher correlation coefficient (R^2^) values and best fitted the Langmuir adsorption isotherm model for all the cases. The information regarding the favorability of the adsorption is determined by the dimensionless constant separation factor or equilibrium parameter, R_L_, which is given by the equation,

8 $${{\rm{R}}}_{{\rm{L}}}=\frac{1}{1+b{C}_{0}}$$

where, b (L/mg) and C_o_ (mg/L) are the Langmuir adsorption constant and the initial dye concentration, respectively. For an adsorption process to be favorable, the R_L_ value must lie between 0 and 1 (0 < R_L_ < 1). For an irreversible process, R_L_ = 0 and for an unfavorable process, R_L_ > 1. In this study, the calculated R_L_ values are in the range of 0 and 1 at different initial dye concentrations of various dyes which indicate that the adsorption of the dyes over NiFeTi2 LDH is a favorable process.

**Table 3:** Isotherm parameters from isotherm study.

Dye	Langmuir model			Freundlich model		
	q_m_ (mg/g)	b (L/mg)	R^2^	K_f_ (mg/g) (L/mg)^1/n^	n	R^2^
MO	32.616	0.3785	0.967	9.379	2.24	0.832
CR	29.970	2.7898	0.991	18.00	4	0.803
MB	29.940	0.5099	0.984	9.484	2.24	0.817
OG	39.952	0.5399	0.903	13.33	2	0.734

**Figure 11:**
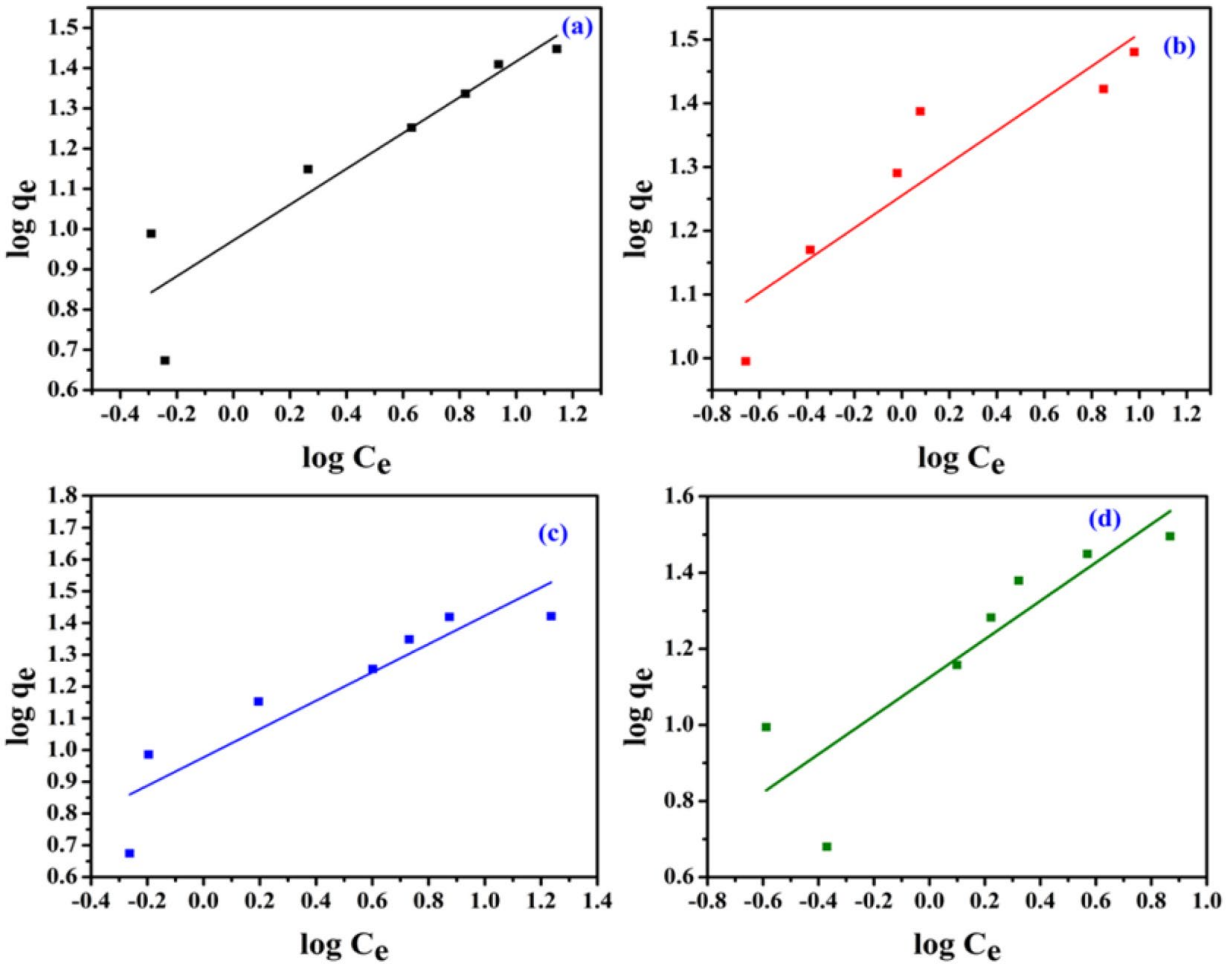
Isotherm plots (Freundlich model) for (a) MO, (b) CR, (c) MB and (d) OG adsorption (V_solution_ = 10 mL, adsorbent amount = 20 mg, T = 25 °C, pH = 7.35).

**Figure 12:**
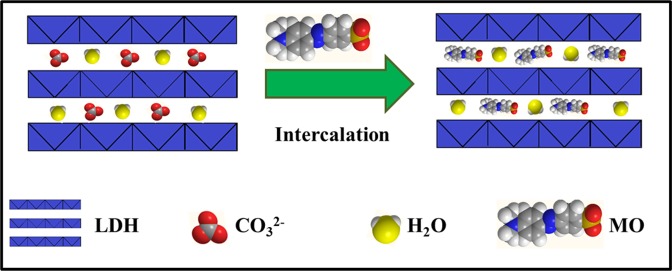
Schematic illustration of adsorption of MO on NiFeTi2 LDH.

The linear form of the Freundlich adsorption isotherm is represented by

9 $${{\rm{logq}}}_{{\rm{e}}}=\frac{1}{n}\,\log \,{{\rm{C}}}_{{\rm{e}}}\,-\,\log \,{{\rm{k}}}_{{\rm{f}}}$$

where, k_f_ and n gives the value of the Freundlich constants. The intercept and the slope of the linear plot of log q_e_ versus log C_e_ (Fig. 11) gives us the value of k_f_ and n, respectively and is shown in Table 3. All the correlation coefficient (R^2^) values are relatively lower than that of Langmuir isotherm. All the n values lie within 1–10, which indicates that the adsorption process is the favorable one. All the results indicate that the surface of NiFeTi2 LDH is homogeneous and follows monolayer uptake mechanism (Fig. 12).

**Figure 13:**
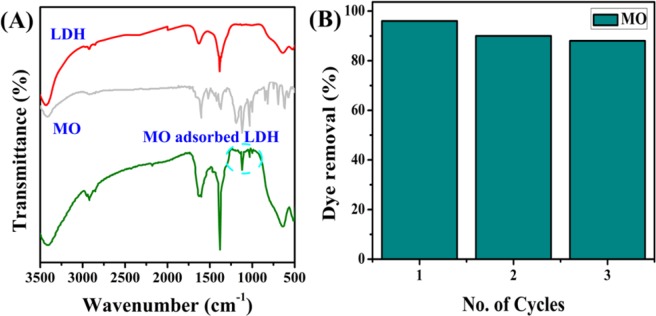
(**A**) FTIR spectra of pure NiFeTi2 LDH, pure MO and adsorbed MO, (**B)** Multicyclic adsorption of MO over NiFeTi2 LDH (V_solution_ = 10 mL, C_0_ = 20 ppm, adsorbent amount = 20 mg, T = 25 °C, pH = 7.35).

To study the adsorption mechanism, FTIR spectra of pure NiFeTi2 LDH, MO and adsorbed MO over NiFeTi2 LDH were compared and are shown in Fig. 13(A). Creation of new bands in the case of MO adsorbed LDH in the wavenumber range of 1000–1300 cm^−1^, evidences the adsorption of MO over the surface of NiFeTi2 LDH. The bands appearing around 1022 and 1120 cm^−1^ could be assigned to the vibration of the –SO_3_^−^ group and 1,4-substituent of the benzene ring of MO dye, respectively^49^. The bands observed at 1176 cm^−1^ and at 1610 cm^−1^ could be attributed to the stretching vibration of C-N and C=C of the benzene ring of MO respectively. Hence, we can conclude that MO is adsorbed to the surface of NiFeTi2 LDH.

The reusability test of NiFeTi2 LDH was carried out and is illustrated in Fig. 13(B). Before carrying out the reusability test, desorption study was performed first, by suspending the used NiFeTi2 LDH in the solution of Na_2_CO_3_ and stirring it for next 24 h. After 24 h, the adsorbent was collected by centrifugation process, washed with decarbonated water and dried at 80 °C for 12 hours. The recovered adsorbent was again subjected for multi-cycles of adsorption for MO under similar reaction conditions. The recovered NiFeTi2 LDH showed dye removal efficiency of 96, 90 and 88% for three successive cycles. Thus, it can be concluded that NiFeTi2 LDH could be considered as a potential adsorbent for complete elimination of organic dyes from contaminated wastewater.

### Biocompatibility

**Figure 14:**
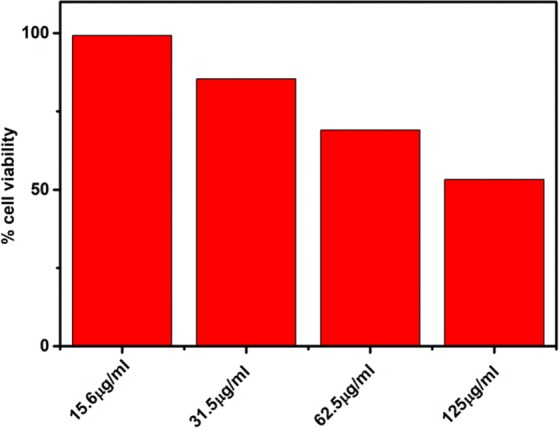
Biocompatibility analysis on MHS cell lines.

The result shows that NiFeTi LDH are biocompatible in nature. At a concentration of 15 μg/ml 100% cells are viable while on increasing the concentration the viability of cells decreases, but at a higher concentration of 125 μg/ml more than 50% cells remain viable (Fig. 14). So we can conclude that NiFeTi LDH is biocompatible in nature^56^.

## Conclusion

In summary, we have successfully synthesized NiFeTi LDH using the hydrothermal method and the resulting material was successfully employed in the adsorptive removal of MO, CR, MB and OG from aqueous solutions. Anionic dyes such as MO (97.44%), CR (98.63%), MB (96.81%) and OG (98.71%) showed comparatively greater adsorption than cationic dyes, MeB (22.81%) and RhB (8.5%) over NiFeTi2 LDH at pH = 7.35. NiFeTi2 gave the best dye removal efficiency (approx. 100% removal in 6 min) with greater adsorption capacity and higher kinetic rate constant. Adsorption equilibrium was attained only within 6 min at T = 25 °C. Pseudo-second order kinetic equation and Langmuir equation perfectly describes the adsorption equilibrium data. The dye adsorption over the LDH surface involved a physisorption process through hydrogen bonding. The reusability of the LDH for multicyclic adsorption process was also explored leading to the conclusion that NiFeTi LDH can be considered as a fast and efficient adsorbent for the removal of anionic dyes from contaminated water. The biocompatibility studies carried on MHS cell lines concluded the non-toxic behaviour of NiFeTi LDH.

## Supplementary information

A new biocompatible ternary Layered Double Hydroxide Adsorbent for ultrafast removal of anionic organic dyes

## Supplementary information

is available this paper at 10.1038/s41598-019-52849-4.
