# Supplementary material for: A new biocompatible ternary Layered Double Hydroxide Adsorbent for ultrafast removal of anionic organic dyes
Source: Sci Rep. 2019 Nov 7;9:16225. doi: 10.1038/s41598-019-52849-4 (PMC6838081; doi:10.1038/s41598-019-52849-4)
Supplement: Supplementary file 1 — A new biocompatible ternary Layered Double Hydroxide Adsorbent for ultrafast removal of anionic organic dyes [file 41598_2019_52849_MOESM1_ESM.pdf]

Supplementary Information

A new biocompatible ternary Layered Double Hydroxide Adsorbent for ultrafast removal of anionic organic dyes

Garima Rathee[1], Amardeep Awasthi[1], Damini Sood[1], Ravi Tomar[1], Vartika Tomar[1] and Ramesh Chandra[1,2]*

Author affiliations:

1- Drug Discovery & Development Laboratory, Department of Chemistry, University of Delhi, Delhi-110007, India.

2- Dr. B. R. Ambedkar Centre for Biomedical Research University of Delhi, Delhi-110007, India.

***Correspondance:**

Prof. Ramesh Chandra, Drug Discovery and Development Laboratory,
Department of Chemistry, University of Delhi, Delhi-110007, India.
E-mail: rameshchandragroup@gmail.com

S1. Structure of the test Dyes

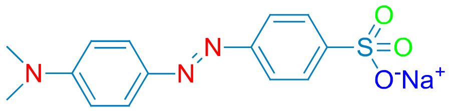

Fig. Sa. Structure of Methyl Orange

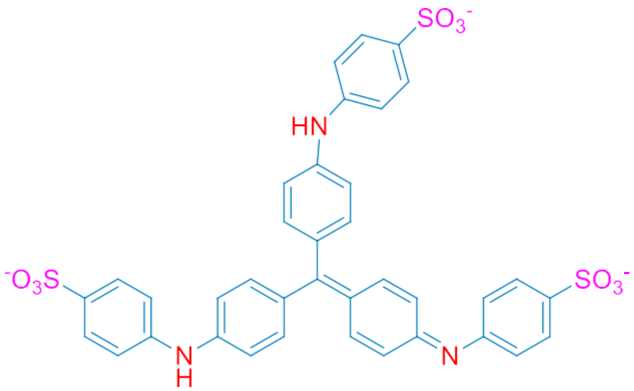

Fig. Sb. Structure of Methyl Blue

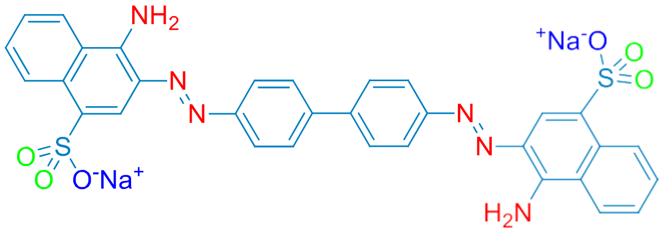

Fig. Sc. Structure of Congo Red

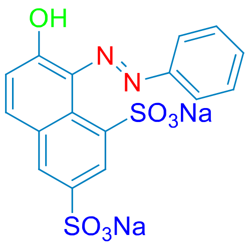

Fig. Sd. Structure of Orange G

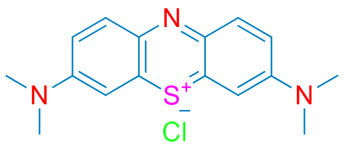

Fig. Se. Structure of Methylene Blue

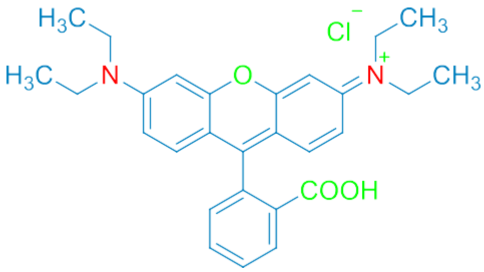

Fig. Sf. Structure of Rhodamine B

S2. Scheme of Ni/Fe/Ti LDH synthesis:

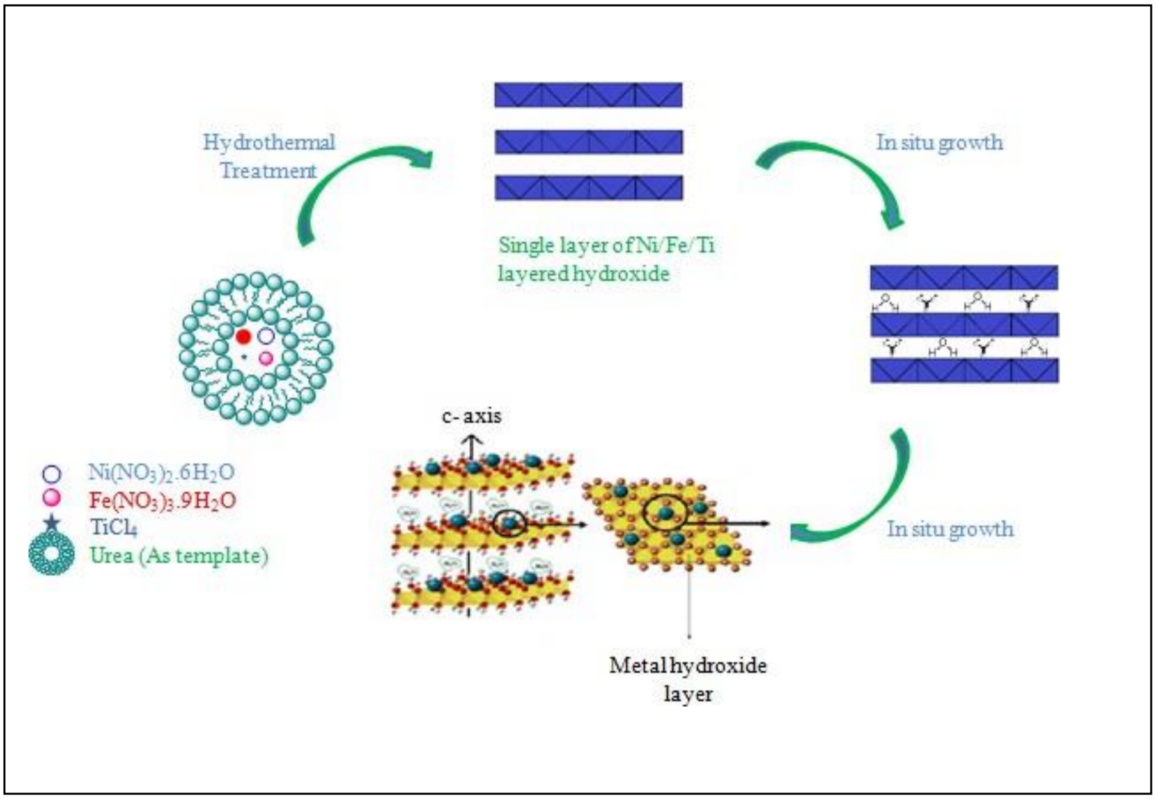

TABLE S1. X-ray diffraction parameters of Ni/Fe/Ti2 LDH:

h	k	l	*2θ/degree*	d spacing/nm
0	0	3	11.12	0.79
0	0	6	22.33	0.39
0	0	9	34.31	0.26
-	-	-	16.16	0.55
1	1	0	25.09	0.35
1	0	1	36.45	0.26
-	-	-	38.524	0.24
0	1	8	46.825	0.19

TABLE S2. ICP results of synthesized materials:

Sample	Ni (ppm)	Fe (ppm)	Ti (ppm)
**NiFeTi1**	0.73	122.66	27.30
**NiFeTi2**	127.64	10.44	51.16
**NiFeTi3**	79.67	30.30	62.55
**NiFeTi4**	6.57	84.22	46.03
**NiFeTi5**	141.23	0.22	67.42

S3. Characterization of synthesized materials:

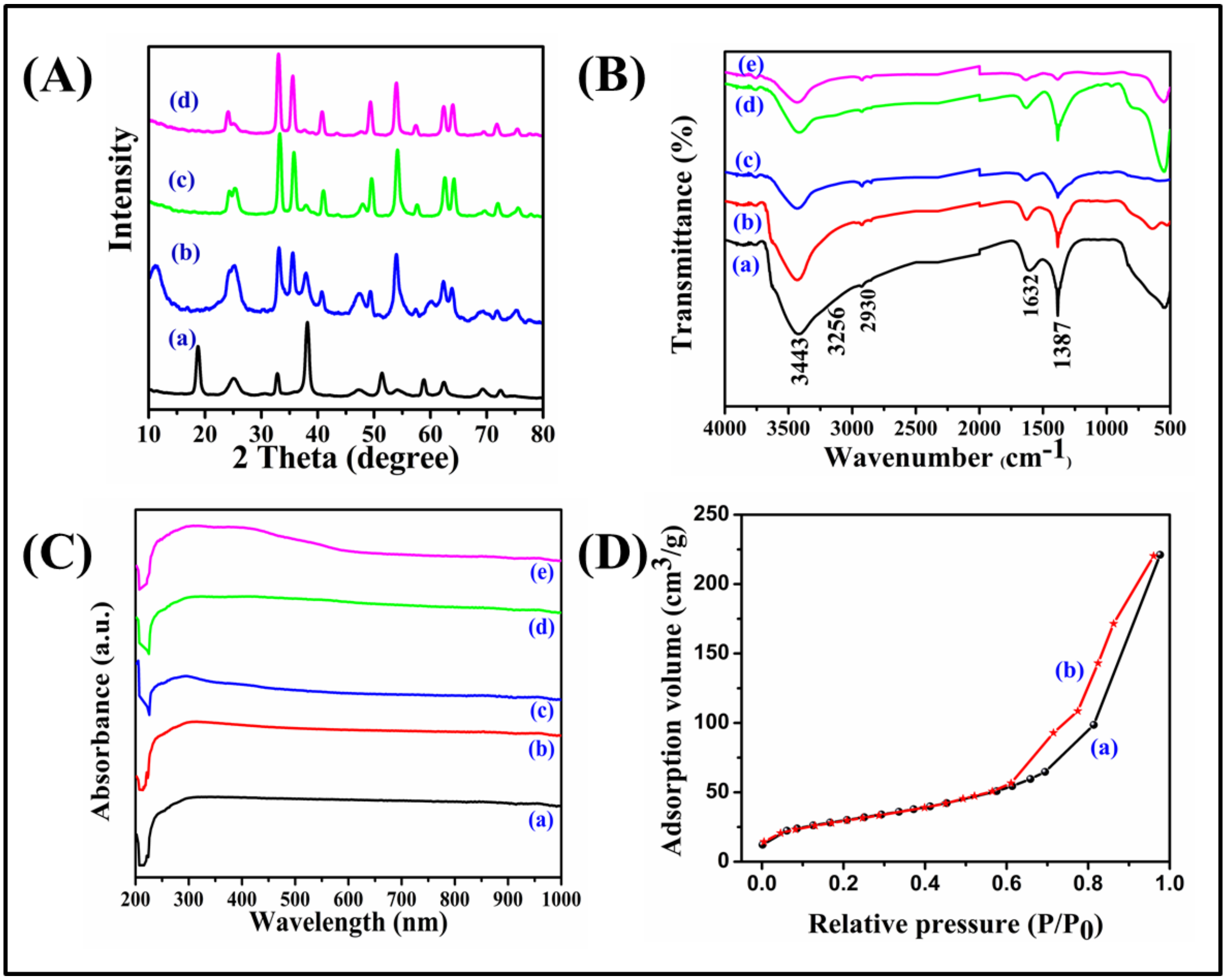

Figure S3a. **(A)** XRD Patterns of synthesized materials [(a) NiFeTi1 LDH, (b) NiFeTi3 LDH, (c) NiFeTi4 LDH, (d) NiFeTi5 LDH], **(B)** FTIR spectra of LDHs [(a) NiFeTi1 LDH, (b) NiFeTi2 LDH, (c) NiFeTi3 LDH, (d) NiFeTi4 LDH, (e) NiFeTi5 LDH], **(C)** UV-vis spectroscopy of LDHs [(a) NiFeTi1 LDH, (b) NiFeTi2 LDH, (c) NiFeTi3 LDH, (d) NiFeTi4 LDH, (e) NiFeTi5 LDH], **(D)** (a) N_2-adsorption and (b) N_2 -desorption of NiFeTi2 LDH.

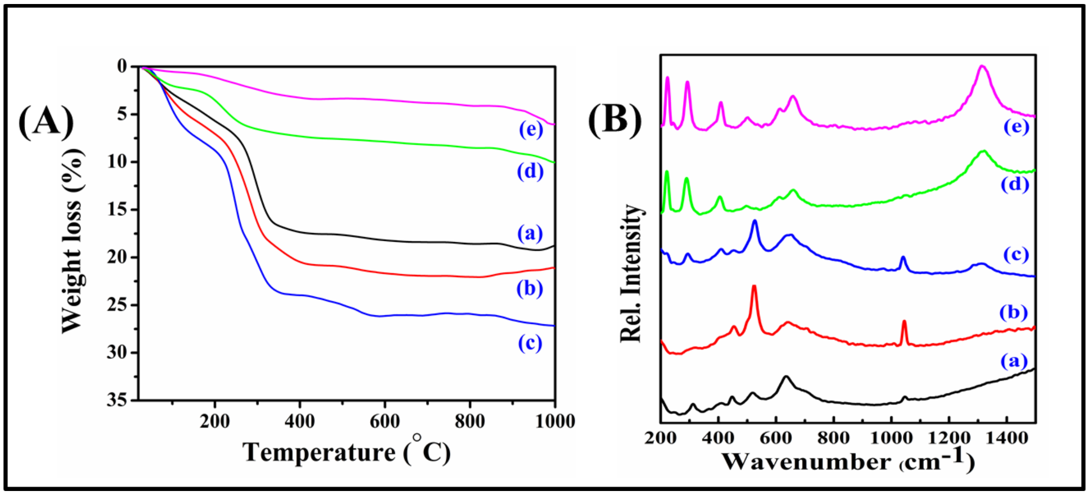

Figure S3b. **(A)** TGA spectra of LDHs [(a) NiFeTi1 LDH, (b) NiFeTi2 LDH, (c) NiFeTi3 LDH, (d) NiFeTi4 LDH, (e) NiFeTi5 LDH], **(B)** Raman spectra of LDHs [(a) NiFeTi1 LDH, (b) NiFeTi2 LDH, (c) NiFeTi3 LDH, (d) NiFeTi4 LDH, (e) NiFeTi5 LDH].

S4. Adsorption spectra of various dyes adsorbed on NiFeTi2 LDH:

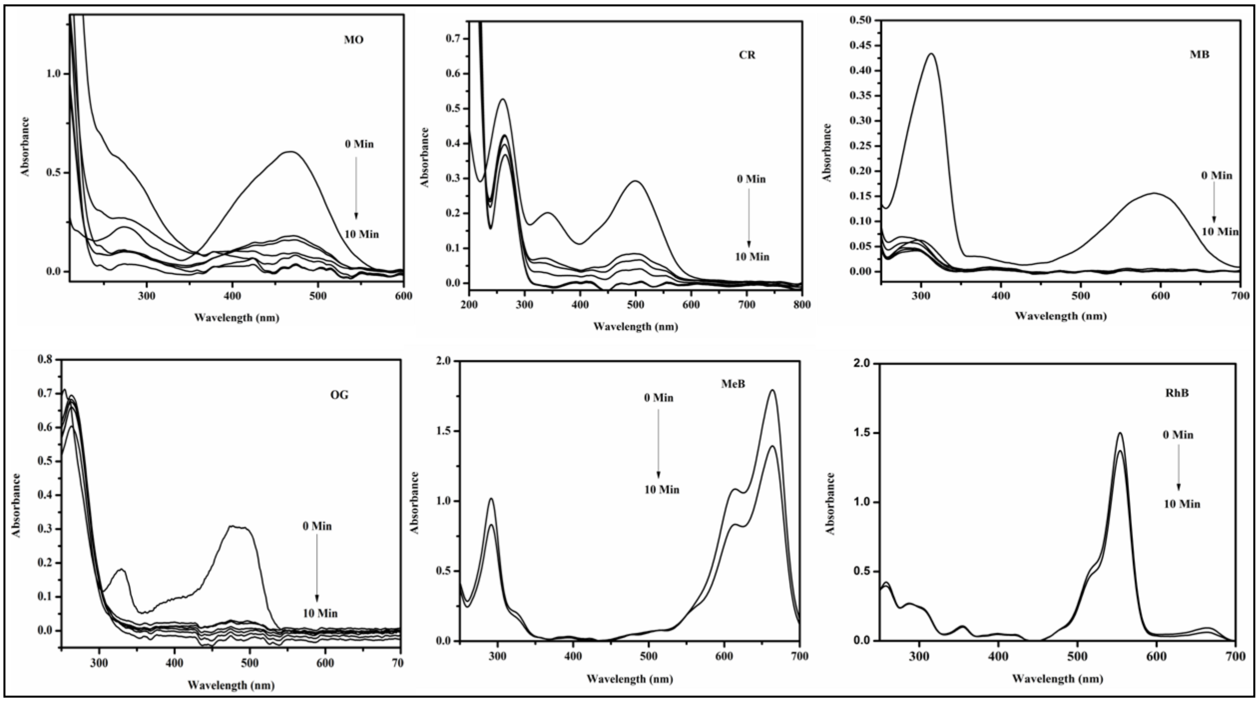

Figure S4. UV-visible spectra of time dependent adsorption of various dyes over NiFeTi2 LDH.

Legends for tables and figures:

TABLE S1. X-ray diffraction parameters of Ni/Fe/Ti 2 LDH:
TABLE S2. ICP results of synthesized materials:

Figure S3a. **(A)** XRD Patterns of synthesized materials [(a) NiFeTi1 LDH, (b) NiFeTi3 LDH, (c) NiFeTi4 LDH, (d) NiFeTi5 LDH], **(B)** FTIR spectra of LDHs [(a) NiFeTi1 LDH, (b) NiFeTi2 LDH, (c) NiFeTi3 LDH, (d) NiFeTi4 LDH, (e) NiFeTi5 LDH], **(C)** UV-vis spectroscopy of LDHs [(a) NiFeTi1 LDH, (b) NiFeTi2 LDH, (c) NiFeTi3 LDH, (d) NiFeTi4 LDH, (e) NiFeTi5 LDH], **(D)** (a) N_2-adsorption and (b) N_2 -desorption of NiFeTi2 LDH.

Figure S4. UV-visible spectra of time dependent adsorption of various dyes over NiFeTi2 LDH.
